# Agrobacterium-Mediated Transformation of Tomato Elicits Unexpected Flower Phenotypes with Similar Gene Expression Profiles

**DOI:** 10.1371/journal.pone.0002974

**Published:** 2008-08-13

**Authors:** Yi-Hong Wang, Michael A. Campbell

**Affiliations:** School of Science, Behrend College, Penn State University, Erie, Pennsylvania, United States of America; Massachusetts General Hospital, United States of America

## Abstract

**Background:**

Genetic transformation mediated by *Agrobacterium tumefaciens* is known to cause unexpected phenotypes. Mutations of a specific set of homeotic genes can result in alterred floral structure.

**Methodology/Principal Findings:**

Previously we identified two genes (*LeTGA1* and *SOLly GLB1*) induced by nutrient availability in tomato. To further elucidate their function, we sought to knock out the genes using antisense RNAi. When antisense constructs for the two different tomato genes were each transformed into Micro-Tina tomato plants, one primary transformant with similar mutant flower phenotypes was identified from transformation of each construct. Microarray analysis shows that a similar set of genes were up- or downregulated in both mutants. Sequencing of insertion sites indicates that each is inserted into a repetitive region which could impact expression of affected genes but direct alteration of floral homeotic gene sequences was not detected.

**Conclusion:**

This is the first report that dominant flower mutations could be caused by genetic transformation designed to knock out two nutrient stress related genes.

## Introduction

For the last two decades, economically important plants have been genetically transformed for longer shelf life, improved nutritional value, enhanced herbicide tolerance, microbial/insect resistance, and tolerance to various severe environmental stresses [1]. However, when a plant is transformed with a transgene, unexpected and undesirable phenotypes may be produced [2], [3], [4].

Unexpected and undesirable phenotypes are frequently encountered as a result of plant transformation [2], [3], [4]. The reasons for the occurrence of unexpected phenotypes abound. First of all, a transgene could insert into, or adjacent to, plant genes and decrease or increase their expression [5]. Secondly, transformation oculd induce chromosome rearrangements such as deletion [5]–[8], translocation [9]–[14], and inversion [15] during transgene insertion. Finally, transgene insertion is not a precisely controlled process [16]–[17] which could be the reason that transgenic plants with unexpected phenotypes are generated in the first place.

Previously, two tomato (*Solanum lycopersicum*) genes (*LeTGA1* and *SOLly GLB1*) induced by nutrient stress treatments were identified using cDNA arrays [18]–[19], which putatively play a role in plant mineral nutrition uptake or utilization. When antisense constructs for the two genes were transformed into tomato plants, one dominant flower mutant was identified from transformation of each construct. While flower structural changes can be caused by mutations in the MADS-box gene family [reviewed in 20]–[22], it is unexpected that antisense to two nutrient stress induced genes would cause mutation in flower structure. It is possible that the mutations could be induced by the transformation process itself [2], [3], [4]. In this paper, we describe the two unexpected tomato flower mutants produced from transformation mediated by *Agrobacterium tumefaciens*.

## Results and Discussion

### Unexpected tomato flower mutants

**Figure 1:**
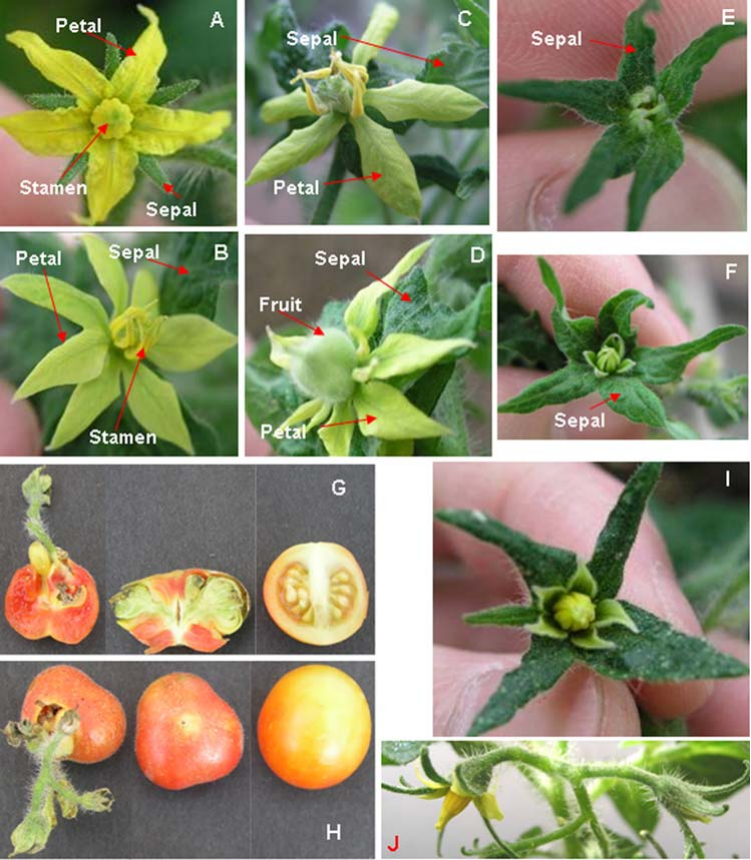
Late flowers from *LS1* (B, C), *LS3* (D), early flowers from *LS1* (E), *LS3* (F) and wild type Micro-Tina (A). These are late flowers that set parthenocarpic fruits (C and D). There were only 1–3 such flowers in each mutant. All other flowers are small and never develop large petals although they do have enlarged sepals. Vascular veins on the petals show a different pattern than the wild type as well. G–H: *LS1* (left and center) and wild type (right) fruits. A shoot with flower buds grows out of one fruit (right). I–J: A control mutant showing slightly enlarged sepals.

To understand the function of a tomato leucine-zipper transcription factor *LeTGA1* [18] and a nonsymbiotic hemoglobin *SOLly GLB1* [19] in plant nutrient uptake/utilization, antisense constructs for the genes were made to generate knockout mutants using the binary vector pBI121 [23]–[24] which has been widely used in tomato transformation [i.e. 25], –[26]. Utilizing *Agrobacterium*-mediated transformation, we generated 78 primary transformants for *LeTGA1* knockout and 130 primary transformants for *SOLly GLB1* knockout. Although majority of the transformants were not notably different from nontransformants, we did find two transgenic tomato plants that have unexpected flower phenotypes with leaf-like sepals (named *L*eafy *S*epals or *LS1* and *LS3*; Figure 1). *LS1* was identified among 78 potential transgenic antisense *LeTGA1* plants and *LS3* was identified among 130 potential transgenic antisense *SOLly GLB1* plants. *LS1* and *LS3* flowers are phenotypically similar to each other (see also Microarray Analysis). The mutants have leafy sepals (Figure 1B–1F) and apparently normal-sized petals/stamens in some late flowers but miniaturized petals/stamens in all early flowers on a truss (Figure 1E, 1F). Petals in the late flowers with normal-sized petals/stamens /carpels that produced fruits (Figure 1H) resemble leaves in terms of vascular vein patterns (Figure 1B, 1C). Another mutant with slightly enlarged sepals is used as a control (Figure 1I, 1J). All mutants are sterile because of alterations in the floral structure. However, the mutants do produce a few parthenocarpic fruits that are structurally different from control fruits. The mutant fruits lack locules and have no seeds, in addition to ectopic shoots growing out of the fruit (Figure 1G). It has been reported that antisense *TM29* (a *SEPALLATA* homolog) tomato mutants also exhibits ectopic shoot growth from fruit but these mutants have different flower morphology [27]. The mutant phenotypes were maintained after one generation of clonal propagation and the plants died before further propagation. These are dominant mutants because the phenotypes appeared in the primary transformants (T_0_) of a selfing plant [28].

### Sequencing of insertion site

**Figure 2:**
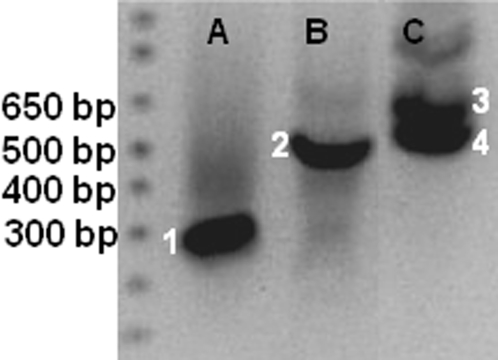
PCR amplified T-DNA right border/plant junction bands using genome walk procedure [Siebert et al. 1995]. Lanes A-*LS1*/*Dra*I; B-*LS1*/*Eco*RV; C- *LS3*/*Stu*I. Each numbered band is cloned and sequenced. Bands 1 and 2 are identical so only sequence from 2 is used. But 3 and 4 are different, probably representing two different insertions in *LS3*. Sizes of molecular weight marker are indicated to the left.

**Figure 3:**
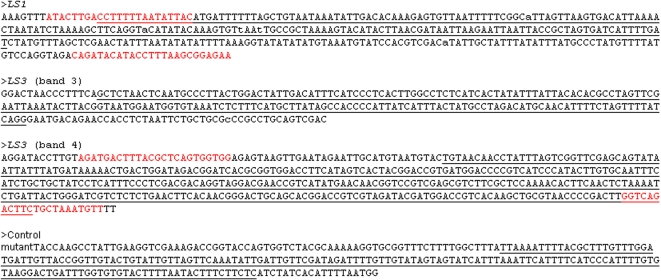
Insertion sequence from *LS1*, *LS2* and a control plant. Sequences in red for *LS1* and *LS3* (band 4) are PCR primers designed to detect the insertion sequence in wild type tomato genome. Underlined sequences are those that match tomato genomic DNA in GenBank and are used to generate data for Table 1.

To find out what could cause such unexpected phenotypes, we sought to identify the sequence of the insertion sites through genome-walk as described by Siebert et al. [29]. Genome walk PCR produced a single band for *LS1* (bands 1 and 2), two bands for *LS3* (bands 3 and 4), suggesting that there may be two insertions in *LS3* and one in *LS1* (Figure 2). These bands were cloned, sequenced and searched against GenBank sequences. Sequencing results showed that bands 1 and 2 are identical; but 3 and 4 are different and that none has an exact match in GenBank. Among the four sequences, band 2 (*LS1*) is partially matched by BI208052 (78% identity) which shares a very low similarity to DNA primase (YP_287459). Band 3 partially matches CV967117 with 75% identity which is 29% identical to the heat shock protein 33. Band 4 sequence is 83% identical to an EST (DB711192) which is not similar to any protein in GenBank. These sequences are presented in Figure 3. As a control, the insertion site in an additional mutant (Figure 1I, 1J) with slightly enlarged sepals (but equally sterile) which contains one insertion was also sequenced (Figure 3 and Table 1). The number of sequence similarity hits along the 12 chromosomes is presented in Table 1. Bands 2, 3 and 4 sequences identified most hits in chromosomes 4, and 8, suggesting that *LS1* and *LS3* insertion sites contain some repetitive sequences (Table 1). Bands 3 and 4 also share similarity (76% and 82% identity) with a retrotransposon Tork3 (accession number EU105454) coding and long terminal repeats regions, respectively; but the significance of this is not clear. In contrast, the control plant which was generated also through antisense *SOLly GLB1*does not seem to be inserted in a repetitive region (Table 1 and Figure 3). To confirm the presence of the insertion sequence identified in the mutants, PCR primers were designed (See Figure 3) and the target regions were detected in wild type Micro-Tina genomic DNA (data not shown). But based on current data, it is difficult to assign exact insertion sites before more genomic sequencing information is available because none of the insertion sequences including control has the exact match to tomato genomic sequences in GenBank. We can only conclude that sequences at insertion sites for *LS1* and *LS3* are repetitive and the implication of this can only be speculated.

**Table 1:** Number of insertion sequence matchs in the 12 tomato chromosomes.

Chrom	1	2	3	4	5	6	7	8	9	10	11	12
*LS1*	2	37		23	7			21	2		1	4
*LS3*-3		2	3	17			1	16	1	2		2
*LS3*-4		10	10	45			3	46	2	5		3
Control		2		1								1

Note: When a match is counted, the homology between the insertion sequence and tomato genomic sequence usually ranged between 66–88% identity. See Figure 3 for sequence that matches the tomato genomic regions.

### Microarray Analysis

**Figure 4:**
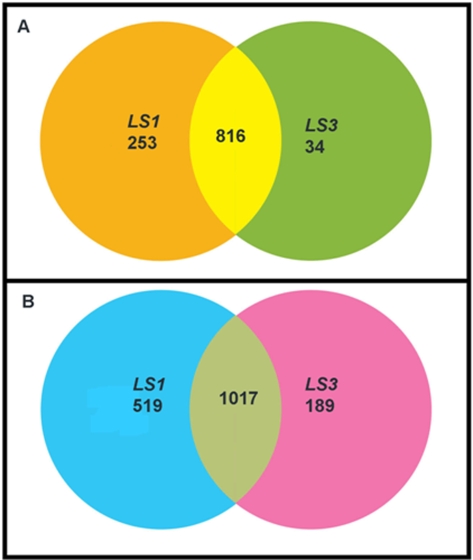
VENN diagram showing the number of cDNAs that exhibit a two-fold change in expression in *LS1* and *LS3* mutants compared to wild-type plants. The down-regulated cDNAs are diagrammed in Panel A and the up-regulated cDNAs are diagrammed in Panel B. In both diagrams, the area of overlap indicates the number of cDNAs which are similarly regulated in both *LS1* and *LS3* mutants.

**Figure 5:**
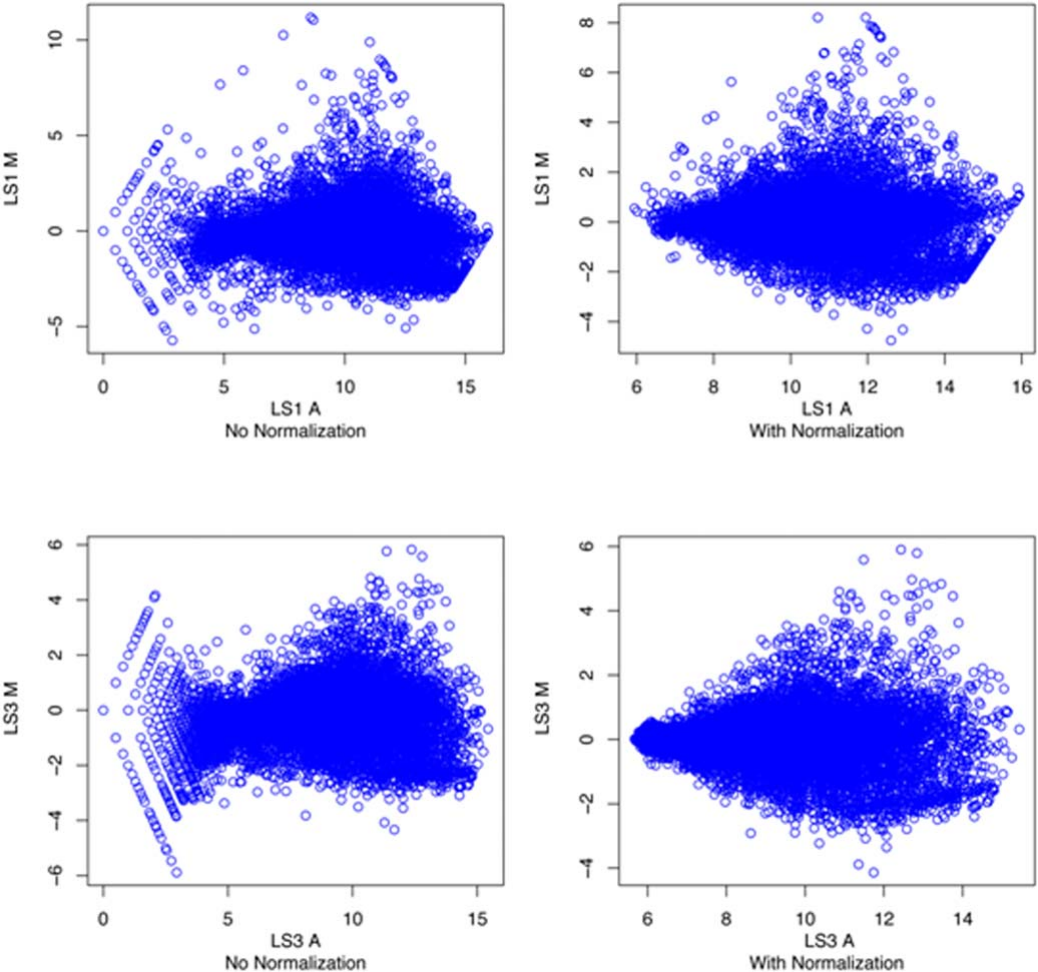
MA plots of microarray data from *LS1* and *LS3* tomato mutants before and after loess normalization.

Expression profiles were determined for *LS1* and *LS3* using a two-color tomato microarray. The majority of cDNAs that exhibit a two-fold change in expression were common to both tomato mutants (Figure 4). This commonality was found to be associated with both up-regulated and down–regulated cDNAs. The *LS1* mutant exhibited a greater number of cDNAs that varied by more than two-fold but there was also a greater range in M values [M = log_2_(c3/cy5)] for that array data (Figure 5). The M values for all significant spots following normalization can be found in Supplement Table S1.

A cDNA (SGN-U147816) exhibits strong up-regulation in both *LS1* and *LS3* (Table 2) and it has 90% identity to the translational product encoded by the gene AtMYB21 from *Arabidopsis thaliana*. AtMYB21 is a myb domain transcription factor that is predominantly expressed in flowers [30]. Another cDNA (SGN-U149060) encoding a ERF-4 (formerly EREBP-4) homolog showed a large decrease in expression in both mutants. ERF-4 is a DNA binding protein that recognizes a GCC-box element [31] and functions as a repressor of gene expression [32]–[33]. The down regulation of the tomato homolog for ERF-4, a putative stress-induced transcription factor, may explain part of the high degree of gene expression similarity for down regulated transcripts in both *LS1* and *LS3*. However, it is not clear what causes the down-regulation of the gene. In addition, flower development related genes [reviewed in 20]–[22] such as *APETALA1* (*AP1*, an A class gene) and *PISTILLATA* (*PI*, a B class gene) were affected in both mutants. While the *PI* homolog is upregulated, the *AP1* homolog is downregulated in these mutants (Table 2). In Arabidopsis, overexpression of *PI* partially converts sepals to petals [34] while mutation in *AP1* partially converts flowers into inflorescence shoots [35]. From microarray data, it is clear that the mutant phenotype was caused by change in expression of flower development related genes and that both mutants show similar expression profiles (Figure 4).

**Table 2:** Up- and down-regulated flower-related genes in both *LS1* and *LS3* mutants in tomato.*

ID Number	Arabidopsis gene homolog	Function	*LS1*	*LS3*
Upregulated genes				
SGN-U149889	*PISTILLATA* (*PI*)	Floral homeotic gene encoding a MADS domain transcription factor. Required for the specification of petal and stamen identities.	2.83	2.33
SGN-U148351	*YABBY1*	Transcriptional regulator involved in abaxial cell type specification in leaves and fruits.	3.07	2.21
SGN-U146520	*ARABIDOPSIS MEI2-LIKE PROTEIN 1*	positive regulation of meiosis, meristem development	3.53	2.49
SGN-U144827	bHLH transcription factor	Causes unfertilized ovules even with normal pollen tube attraction when mutated	3.91	3.06
SGN-U147816	*AtMYB21*	Flower-specific, regulated by COP1 which is involved in light response	4.93	3.74
Downregulated genes				
SGN-U152065	*APETALA1* (*AP1*)	Floral homeotic gene encoding a MADS domain protein homologous to SRF transcription factors. Specifies floral meristem and sepal identity. Required for the transcriptional activation of AGAMOUS. Interacts with LEAFY.	−3.12	−1.98
SGN-U150244	MADS-box protein (*AGL20*)	Controls flowering and is required for CO to promote flowering. It acts downstream of FT.	−2.75	−2.17
SGN-U143668	*NAC2*	Expressed in floral primordia and upregulated by AP3 and PI. Its expression is associated with leaf senescence.	−2.62	−2.28
SGN-U146998	MADS-box protein (*AGL24*)	Represses *FT* expression via direct binding to the *FT* promoter.	−2.58	−1.06

* The numbers in the right two columns are M value. A value of 1.0 corresponds to a 2-fold increase in expression compared to the wild type and a −1.0 means a 2-fold decrease compared to the wild type.

It seems likely that the transformation-related changes in the genome may be responsible for this intriguing phenotype. However, it can not be a result of transgene expression because they are antisense constructs for two different genes which do not share any similarity (data not shown). Previously, it has been shown that both *LeTGA1* and *SOLly GLB1* are induced at transcription level by nutrient availability [18]–[19]. Both genes may negatively impact plant growth if knocked down but may not necessarily play a role in nutrient acquisition [Y.-H. Wang and L.V. Kochian, unpublished results]. RT-PCR to check expression of the two genes did not find any of the ten plants with noticeable decrease of transcript level (data not shown). Therefore, it is not likely that they regulate flower development. This is also because only one of 78 or 130 primary transformants exhibits the flower phenotype. Sequencing of the insertion sites indicates that three insertion sites in the two *LS* mutants contain repetitive sequences while insertion sequence in the control mutant, which does not have the dramatic flower phenotype, is not a repetitive sequence based on GenBank search. This implies that repetitive sequences somehow contributed to the phenotype. It has been suggested that repetitive sequences may serve as either initiators or boundaries for heterochromatin domains [36] which can impact expression of affected genes. In addition, repetitive sequence is positively correlated with methylation [37] which suppresses expression of affected genes. So it is possible that transformation process itself caused changes in the genome that trigger the dramatic phenotype via associated changes in gene expression (see Table 2).

## Materials and Methods

### Plasmid Construct

Antisense constructs were made using the binary vector pBI121 [23]–[24]. For *SOLly GLB1* [19; accession number AY026343] antisense construct, gene coding region was amplified using flanking primers of LeHbSac (5′-GAG CTC CAC GAG AAT CAT CAA TCA TGA GTA G-3′) and LeHbXma (5′-CCC GGG TAC AAG TAT TTT GAA CTG ATG ATC AAT-3′). The resulting PCR product of 618 bp was cloned onto pGEM TA Easy vector (Promega). Selected clones were minipreped, digested with *Sac*I and *Xma*I and cloned into *Sac*I and *Xma*I digested pBI121. For *LeTGA1* [18; accession number AF387785], the gene fragment was amplified with LeTGASac (5′-GAG CTC ATG AAT TCT TCAA CAT ATA CTC-3′) and LeTGAXma (5′-CCC GGG AGT GAG CTA AGA GCA CGA AGA CGT-3′). The fragment was 1087 bp and was cloned into pBI121 as above behind the 35S promoter. BLAST analysis revealed no similarity between *LeTGA1* and *SOLly GLB1*sequences. Both constructs were transformed into *Agrobacterium tumefaciens* strain GV3101 for tomato transformation.

### Tomato transformation

Standard protocol [38] was followed for tomato transformation. Micro-Tina tomato seeds were sterilized and sown on Murashige and Skoog (MS) medium with vitamins. Five to seven day-old cotyledons from the seedlings were cut at the petioles and at the tips. The explants were incubated upside down on MS plates with appropriate vitamins and hormones at room temperature for overnight. *Agrobacterium tumefaciens* GV3101 strain containing a gene construct was cultured on the same day for transformation of these explants the next day. The explants were added to 20 mL of *Agrobacterium* cell and incubated for 15 minutes with periodic shaking. The explants were then returned to their plates upside down, sealed with micropore tape and incubated at room temperature for two days in subdued light. After this, the explants were transferred into regeneration media to allow for regeneration of shoots. As soon as shoots appeared (about 4–8 weeks), they were transferred to rooting medium. After the shoots developed adequate roots, they were transplanted into greenhouse. *LS1*, *LS3*, and the control mutants (Figure 1) were identified when grown in the greenhouse. Since none of the three mutants are fertile, all experiments described in this paper are on T_0_ mutant plants.

### Sequencing of T-DNA right border insertion site

The PCR-based genome walk procedure [29] was used. Tomato genomic DNA was isolated using a Qiagen DNeasy Plant Kit and was digested with a blunt-end restriction enzyme and ligated to an adaptor to create a library of DNA fragments. Adaptor sequences used were 5′-CTA ATA CGA CTC ACT ATA GGG CTC GAG CGG CCG CCC GGG CAG GT-3′ (Ad1) and 5′-P-ACC TGC CC-NH_2_-3′ (Ad2) [29]. PCR was performed on the library using a primer complementary to the adaptor sequence (AP1: 5′-GGA TCC TAA TAC GAC TCA CTA TAG GGC-3′) [29] and a primer specific to the vector DNA sequence [IP1: 5′-CGT TGC GGT TCT GTC AGT TCC-3′; 23]. In the first PCR cycle, primer extension occurred only from the specific PCR primer that binds to the vector sequence in the DNA fragment within the library. Subsequent PCR using nested primers (IP1nest: 5′-GGTTCTGTCAGTTCCAAACG-3′ and AP2: 5′-AAT AGG GCT CGA GCG GC-3′) complementary to the vector and adaptor sequences generated a DNA fragment. Fragment that did not contain a sequence complementary to the specific primer were not amplified. The PCR products were cloned into a TA-cloning vector pGEM-T Easy (Promega) and sequenced using T7 or SP6 primers. Sequencing was performed at the Penn State Nucleic Acid Facility at University Park, PA.

### Nucleic acid isolation and microarray hybridization

Flowers from mutant or wild-type plants were ground to a fine powder in liquid nitrogen. Total RNA was isolated using a Trizol® extraction followed by purification using an affinity column (www.affymetrix.com). The purity of the RNA was determined using a spectrophotometer and integrity was confirmed using gel electrophoresis and visualization of ribosomal bands. RNA samples were labeled using a Superscript Plus Indirect cDNA labeling System (Invitrogen). Hybridization was to a Tom1 tomato cDNA microarray which was the only tomato microarray available at the time (http://bti.cornell.edu/CGEP/CGEP.html). Arrays were hybridized and washed according to the procedures outlined by DeRisi (http://derisilab.ucsf.edu/microarray/protocols.html) using a Biosciences Lucidea Slidepro Hybridizer (Amersham). Arrays were scanned at 532 and 635 nm using a Genepix 4000B (Axon Instruments) and gpr files were created using Genespring GX 7.3 software (Agilent Technologies). Expression levels were determined for the average across the arrays in two different replicates. Gpr files were loess normalized using the R Bioconductor package marray (Figure 5). Expression values were determined (M = log2(635nm/532nm) and averaged for duplicate spots. Genes exhibiting two-fold up or two-fold down expression (M≥±1) were selected for additional analysis.

## Supporting Information

Table S1 Microarray data for LS1 and LS3
(1.95 MB XLS)
